# Bayesian model for accurate MARSALA (mutated allele revealed by sequencing with aneuploidy and linkage analyses)

**DOI:** 10.1007/s10815-019-01451-8

**Published:** 2019-06-11

**Authors:** Luoxing Xiong, Lei Huang, Feng Tian, Sijia Lu, Xiaoliang Sunney Xie

**Affiliations:** 1 Peking-Tsinghua Center for Life Sciences (CLS), Academy for Advanced Interdisciplinary Studies, Peking University, Beijing, 100871 China; 2 Biomedical Pioneering Innovation Center (BIOPIC), School of Life Sciences, Peking University, Beijing, 100871 China; 3 Beijing Advanced Innovation Center for Genomics (ICG), Peking University, Beijing, 100871 China; 4 Department of Chemistry and Chemical Biology, Harvard University, Cambridge, MA 01238 USA; 5 Yikon Genomics Co., Ltd., 1698 Wangyuan Road, Building #26, Fengxian District, Shanghai, 201400 China

**Keywords:** MARSALA, PGT-M, Bayesian statistics, Linkage analyses

## Abstract

**Purpose:**

This study is aimed at increasing the accuracy of preimplantation genetic test for monogenic defects (PGT-M).

**Methods:**

We applied Bayesian statistics to optimize data analyses of the mutated allele revealed by sequencing with aneuploidy and linkage analyses (MARSALA) method for PGT-M. In doing so, we developed a Bayesian algorithm for linkage analyses incorporating PCR SNV detection with genome sequencing around the known mutation sites in order to determine quantitatively the probabilities of having the disease-carrying alleles from parents with monogenic diseases. Both recombination events and sequencing errors were taken into account in calculating the probability.

**Results:**

Data of 28 in vitro fertilized embryos from three couples were retrieved from two published research articles by Yan et al. (Proc Natl Acad Sci. 112:15964–9, 2015) and Wilton et al. (Hum Reprod. 24:1221–8, 2009). We found the embryos deemed “normal” and selected for transfer in the previous publications were actually different in error probability of 10^−4^–4%. Notably, our Bayesian model reduced the error probability to 10^−6^–10^−4^%. Furthermore, a proband sample is no longer required by our new method, given a minimum of four embryos or sperm cells.

**Conclusion:**

The error probability of PGT-M can be significantly reduced by using the Bayesian statistics approach, increasing the accuracy of selecting healthy embryos for transfer with or without a proband sample.

**Electronic supplementary material:**

The online version of this article (10.1007/s10815-019-01451-8) contains supplementary material, which is available to authorized users.

## Introduction

There are 6000–7000 monogenic diseases, affecting millions of people [1]. Most of these genetic disorders are severe and effective therapies against them are rare [1]. Because specific mutations for the monogenic diseases are usually heterozygous, couples affected can have healthy embryos that can be selected for implantation through in vitro fertilization(IVF) with PGT-M [2]. On the other hand, IVF embryos also need to be selected against aneuploidy, which is caused by abnormal chromosome numbers and often leads to live birth failure, by preimplantation genetic testing for aneuploidies (PGT-A) [3–5]. To conduct PGT-M and PGT-A at the same time, SNP arrays [6] and next generation sequencing (NGS) [7–11] have been used previously.

In 2015, we reported mutated allele revealed by sequencing with aneuploidy and linkage analyses (MARSALA), an improved method for PGT-M. MARSALA relied on both the linkage analyses and direct sequencing of the targeted mutation sites in one next-generation sequencing run, which offered more reliable performance than previous methods [7].

Linkage analyses deal with the fact that false positive and false negative error rates are non-zero at a particular single-nucleotide variant (SNV) site, relying on the detected SNVs near the causal mutation to deduce whether the disease-carrying allele is present in the embryo [12–14]. The linkage analysis is critical for PGT-M because it significantly reduces the error probability. According to two recent reviews, the error of linkage analysis was reduced from 3 to 4% to 0.4–0.5% [15] for multiplex PCR and 0.3% [16] for Karyomapping. The linkage analysis with MARSALA [7] offered higher precision; however, to the best of our knowledge, the error rates have not been quantified yet.

Our goal is to further reduce the risk to 10^−6^–10^−4^%, because PGT-M patients in European countries alone are about 10,000 per year [17], and even higher and growing number exists in China [18]. In the present work, we used Bayesian statistics to determine the error rate for MARSALA with the data presented in two published papers [7], Haitao Wu et al.]. The Bayesian statistics model is based on the recombination probabilities and SNV error rates at different genome locations.

In addition to limited accuracy, the majority of previous linkage analyses are also limited by a proband sample, which is not always available, particularly in an unhealthy status [6, 7]. Several reports have performed linkage analyses without proband in MARSALA-based PGT-M, an affected embryo or sperm cell was used instead of a proband sample [17, 19,20]. We applied our method to the data using sperm cells as proband [17] and calculated disease-carrying probability for each embryo.

## Materials and methods

### Samples

Sequencing data were taken from our two published studies [7, 17] and reanalyzed. Part of the sequencing data for cases 1 and 2 was from SRP067387 [7]. The study was approved by the Reproductive Study Ethics Committee at Peking University Third Hospital (research license 2014SZ001). In case 1, the father has a family history of hereditary multiple exostoses and suffers from this disease. The affected grandfather, both parents, and 18 embryos were sequenced (Table 1). In case 2, the mother carries an X-linked mutation and her son suffers from hypohidrotic ectodermal dysplasia. The affected born child, both parents, 4 embryos, and their corresponding 8 polar bodies were sequenced (Table 1). Sequencing data for case 3 was from originally published sequencing data [17]. The study was approved by the Research Ethics Committee of the First Hospital of Sun Yat-sen University [2014]134. In case 3, both parents are affected with beta thalassemia and present different mutation sites. Both parents and seven sperm cells were sequenced. All samples were whole-genome amplified (WGA) using MALBAC [21] kit (Yikon Genomics Inc.). After WGA, the causal mutation region was enriched by PCR amplification using specific primers in proximity to the affected area (Table S1). The total product was then sequenced using Illumina Hiseq 2500 with ~ 2× mean genome depth.

**Table 1:** Sample description. case 1 and case 2 are from reference [7], and case 3 is from reference [17]

Case ID	Amplification	Mutation	Disease parent	Proband	Sperm	Polar body	Embryo number	Data source
Case 1	WGS-2×	chr11:69255368 T>G	Father	1	0	0	18	Ref [7]
Case 2	WGS-2×	chrX:44129492 delC	Mother	1	0	8	4	Ref [7]
Case 3	WGS-2×	chr11:5248329 A>G chr11:5427992 delAAAG	Both parents	0	7	0	6	Ref [17]

### Calculating disease-carrying probability

**Fig. 1:**
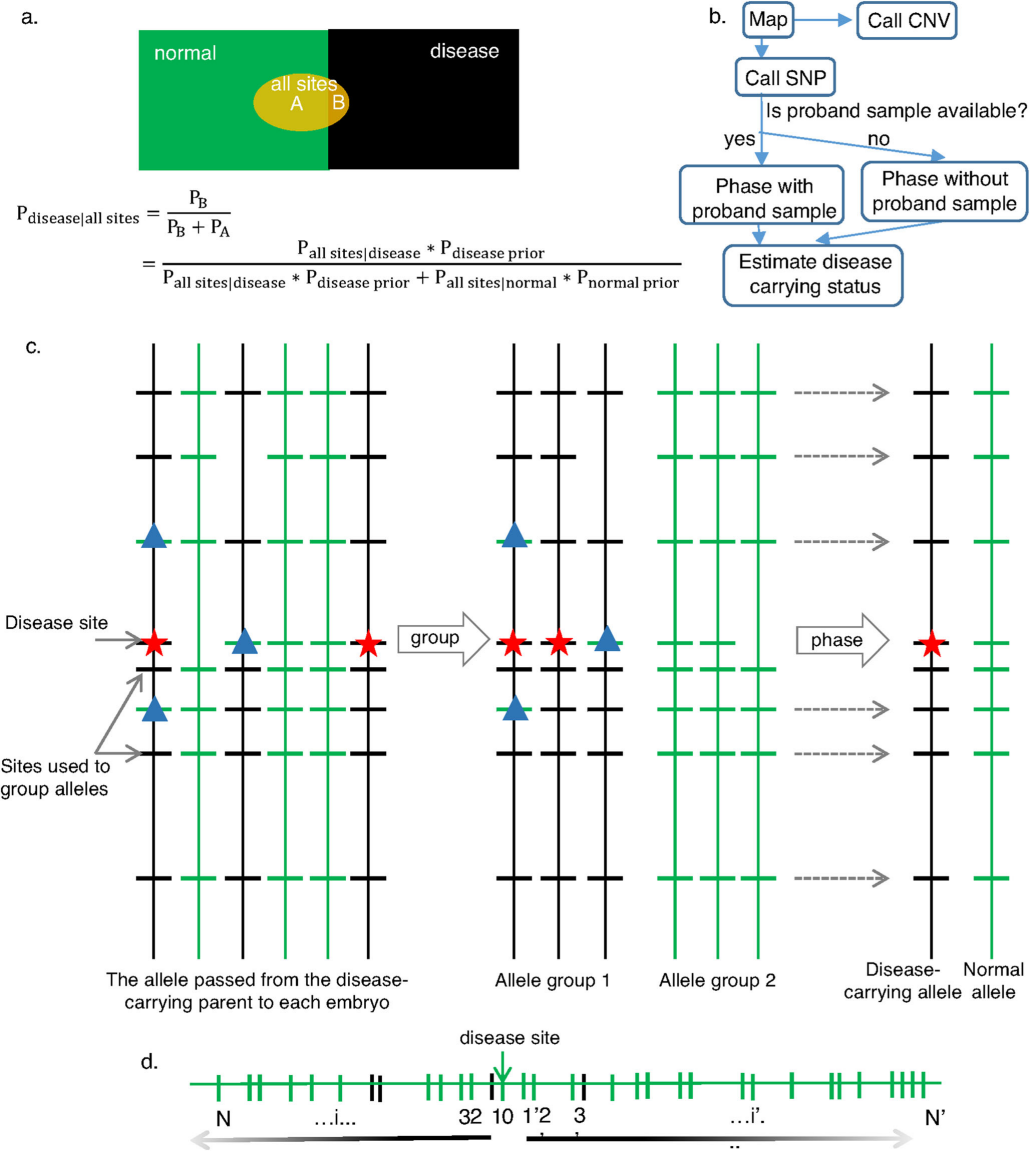
Experimental pipeline of MARSALA and Bayesian model-based linkage analyses. **a** Sketch map of using Bayesian inference to calculate disease-carrying probability. Green box represents all sites of the normal allele for any normal embryo. Black box represents all sites of the disease-carrying allele for any disease-carrying embryo. “all sites” means all of the available linkage sites, which are 1.5 Mb upstream or downstream of the causal mutation site and are derived from the sequencing data, in an embryo. Some of the “all sites” seem to come from the normal allele, which is marked as “A,” while the rest of them seem to come from the disease-carrying allele, which is marked as “B.” According to Bayes’ theorem, the posterior probability *P*_disease|all_ site is calculated from prior probabilities and conditional probabilities, which is composed of recombination ratios and sequencing errors. **b** Analyses pipeline of the Bayesian model-based data analyses. We first map sequence reads to the reference genome hg19, then call CNVs to avoid aneuploidy. Meanwhile, SNPs are called from the mapped data. Afterwards, we phase the disease-carrying allele with proband sample if a proband sample is available, or else we can phase the alleles without proband sample, which is depicted in Fig. 1c. At last, we can calculate the disease-carrying probability for each embryo with the phased disease-carrying allele. **c** Phasing without proband sample. First, deduce the allele passed from the disease-carrying parent to each embryo. Because the disease-carrying parent is heterozygous, these alleles could be grouped into two classes by sites where genotype is available in most of the alleles. One class should be healthy, while the other allele carries causal mutation. The normal allele and the disease-carrying allele are phased based on these two classes. Green represents sites that appear to come from the healthy allele. Black means that the site appears to come from the disease-carrying allele. Sites marked with red star is the disease site. And sites marked with blue triangle are those sites that suffer from sequencing error or mapping error. **d** Sketch map of linkages sites in an embryo

The disease-carrying allele is either phased with similar methods with previous analyses [7] (Fig. 1b, Fig. S1, Supplementary methods) when a proband sample is available, or phased as described in the next section when a proband sample is absent. After phasing the disease-carrying allele, error probability is calculated to estimate an embryo’s disease-carrying status through Bayesian inference. Bayesian inference is a method of calculating posterior probability according to Bayes’ theorem (https://en.wikipedia.org/wiki/Bayesian_inference):

$$ {P}_{\mathrm{H}\mid \mathrm{E}}=\frac{P_{\mathrm{E}\mid \mathrm{H}}\times {P}_{\mathrm{H}}}{P_{\mathrm{E}}}=\frac{P_{\mathrm{E}\mid \mathrm{H}}\times {P}_{\mathrm{H}}}{P_{\mathrm{E}\mid \mathrm{H}}\times {P}_{\mathrm{H}}+{P}_{\mathrm{E}\mid \mathrm{no}\ \mathrm{H}}\times {P}_{\mathrm{noH}}} $$

, where *P*_H ∣ E_ represents the probability of the hypothesis (H) given the evidence (E); *P*_E ∣ H_ means the probability of the evidence (E) if the hypothesis (H) is true. *P*_H_ is the prior probability of the hypothesis, which is the estimated before evidence (E). *P*_E_ is the total probability of evidence (E). And “no H” means the negative side of the hypothesis.

In our case, the evidence (E) is the sequencing data of a proband, parents, and embryos, i.e., the phased disease-carrying allele and genotypes at all sites in the embryos, thus written as “all sites” in the following formula. The hypothesis (H) is that the embryo carries disease. So the probability of the embryo carrying disease given the all sites is written as *P*_disease ∣ all sites_, shortened as *P*_disease_. “no H” means embryo is normal. Then *P*_disease_ can be calculated according to Bayes’ theorem (Fig. 1a) as follows:

${P}_{\mathrm{disease}}=\frac{P_{\mathrm{all}\ \mathrm{sites}\mid \mathrm{disease}}\times {P}_{\mathrm{disease}\ \mathrm{prior}}}{P_{\mathrm{all}\ \mathrm{sites}\mid \mathrm{disease}}\times {P}_{\mathrm{disease}\ \mathrm{prior}}+{P}_{\mathrm{all}\ \mathrm{sites}\mid \mathrm{normal}}\times {P}_{\mathrm{normal}\ \mathrm{prior}}}$, where *P*_disease_ means the probability of the embryo carrying disease given the sequencing data (all sites); *P*_all sites ∣ disease_ means the conditional probability of observing the genotypes at all sites if the embryo carries disease. *P*_disease prior_ is the prior probability of the embryo carrying disease before sequencing data is obtained. The probabilities of “normal,” *P*_all sites ∣ normal_ and *P*_normal prior_, are similar with those of “disease.”

To compute *P*_disease_ for each embryo, we need to calculate the prior probabilities and conditional probabilities. The prior probability, *P*_normal prior_ and *P*_disease prior_, of an embryo carrying disease, or being normal, is 0.5 for both to reflect Mendelian genetics. If there are *N* sites upstream of the causal mutation site and *N*′ sites downstream (Fig. 1d), the conditional probability of *P*_all sites ∣ disease_ and *P*_all sites ∣ normal_ could be computed from upstream and downstream sites as follows:

$$ {P}_{\mathrm{all}\ \mathrm{sites}\mid \mathrm{disease}}={P}_{\mathrm{sites}\ 1\ \mathrm{to}\ N\mid \mathrm{disease}}\times {P}_{\mathrm{sites}\ {1}^{\prime }\ \mathrm{to}\ {N}^{\prime}\mid \mathrm{disease}} $$

$$ {P}_{\mathrm{all}\ \mathrm{sites}\mid \mathrm{normal}}={P}_{\mathrm{sites}\ 1\ \mathrm{to}\ N\mid \mathrm{normal}}\times {P}_{\mathrm{sites}\ {1}^{\prime }\ \mathrm{to}\ {N}^{\prime}\mid \mathrm{normal}} $$

If recombination rates in non-overlapping regions are independent, conditional probability of upstream sites is calculated as follows. Conditional probability of downstream sites is calculated in a similar manner.

$$ {P}_{\mathrm{site}\mathrm{s}\ 1\ \mathrm{to}\ N\mid \mathrm{disease}}={P}_{\mathrm{site}\mathrm{s}\ 1\ \mathrm{to}\ N\mid \mathrm{site}\ 0\ \mathrm{disease}}={P}_{\mathrm{site}\mathrm{s}\ 2\ \mathrm{to}\ N\mid \mathrm{site}\ 1\ \mathrm{disease}}\times {P}_{\mathrm{site}\ 1\ \mathrm{disease}}\times \left(1-{P}_{\mathrm{recom}\ 01}\right)+{P}_{\mathrm{site}\mathrm{s}\ 2\ \mathrm{to}\ N\mid \mathrm{site}\ 1\ \mathrm{normal}}\times {P}_{\mathrm{site}\ 1\ \mathrm{normal}}\times {P}_{\mathrm{recom}\ 01} $$

$$ {P}_{\mathrm{site}\mathrm{s}\ 1\ \mathrm{to}\ N\mid \mathrm{normal}}={P}_{\mathrm{site}\mathrm{s}\ 1\ \mathrm{to}\ N\mid \mathrm{site}\ 0\ \mathrm{normal}}={P}_{\mathrm{site}\mathrm{s}\ 2\ \mathrm{to}\ N\mid \mathrm{site}\ 1\ \mathrm{disease}}\times {P}_{\mathrm{site}\ 1\ \mathrm{disease}}\times {P}_{\mathrm{recom}\ 01}+{P}_{\mathrm{site}\mathrm{s}\ 2\ \mathrm{to}\ N\mid \mathrm{site}\ 1\ \mathrm{normal}}\times {P}_{\mathrm{site}\ 1\ \mathrm{normal}}\times \left(1-{P}_{\mathrm{recom}\ 01}\right) $$

Similarly, conditional probability of any site *i*-1 could be computed from site *i* when *i* < =*N*−1 and *i* > =1.

$$ {P}_{\mathrm{site}\mathrm{s}\ i\ \mathrm{to}\ N\mid \mathrm{site}\ i-1\ \mathrm{disease}}={P}_{\mathrm{site}\mathrm{s}\ i+1\ \mathrm{to}\ N\mid \mathrm{site}\ i\ \mathrm{disease}}\times {P}_{\mathrm{site}\ \mathrm{i}\ \mathrm{disease}}\times \left(1-{\mathrm{P}}_{\mathrm{recom}\ i\left(i-1\right)}\right)+{P}_{\mathrm{site}\mathrm{s}\ i+1\ \mathrm{to}\ N\mid \mathrm{site}\ \mathrm{i}\ \mathrm{normal}}\times {P}_{\mathrm{site}\ i\ \mathrm{normal}}\times {P}_{\mathrm{recom}\ i\left(i-1\right)} $$

$$ {P}_{\mathrm{site}\mathrm{s}\ i\ \mathrm{to}\ N\mid \mathrm{site}\ i-1\ \mathrm{normal}}={P}_{\mathrm{site}\mathrm{s}\ i+1\ \mathrm{to}\ N\mid \mathrm{site}\ i\ \mathrm{disease}}\times {P}_{\mathrm{site}\ i\ \mathrm{disease}}\times {P}_{\mathrm{recom}\ i\left(i-1\right)}+{P}_{\mathrm{site}\mathrm{s}\ i+1\ \mathrm{to}\ N\mid \mathrm{site}\ i\ \mathrm{normal}}\times {P}_{\mathrm{site}\ i\ \mathrm{normal}}\times \left(1-{P}_{\mathrm{recom}\ i\left(i-1\right)}\right) $$

when *i* equals to *N*,

*P*_sites *N* to *N* ∣ site *N* − 1 disease_ = *P*_site *N* disease_ × (1 − *P*_recom *N*(*N* − 1)_) + *P*_site *N* normal_ × *P*_recom *N*(*N* − 1)_, recombination rate *P*_recom *i*(*i* − 1)_ could be computed as follows:

*P*_recom *i*(*i* − 1)_ = *P*_recom in the 1Mb region_ × *P*_distance *i*(*i* − 1)(/Mb)_, *P*_recom in the 1Mb region_ is referred to the recombination rate estimated by deCODE [22]. Notably, PCR product of the causal mutation site and linkage analyses separately estimated the disease-carrying status in previous MARSALA analyses. In Bayesian inference, PCR result of the disease causal mutation site is combined to linkage analyses. The disease site is introduced as a special linkage site, by setting the recombination rate between this special linkage site and the disease site to 0.

*P*_site *i* disease_ and *P*_site *i* normal_ are the probability of site *i* coming from the disease-carrying and the normal allele, respectively. They are calculated by combining the genotype probability generated by GATK [23] of all the family members.

$$ {P}_{\mathrm{site}\ i\ \mathrm{disease}}={\Sigma P}_{\mathrm{disease}-\mathrm{supportive}\ \mathrm{combination}}+\frac{1}{2}{\Sigma P}_{\mathrm{neutral}\ \mathrm{combination}} $$

${P}_{\mathrm{site}\ i\ \mathrm{normal}}={\Sigma P}_{\mathrm{normal}-\mathrm{supportive}\ \mathrm{combination}}+\frac{1}{2}{\Sigma P}_{\mathrm{neutral}\ \mathrm{combination}}$, *P*_disease − supportive combination_ means the probability of the genotype combinations of the parents and embryos, based on which the site appears to come from the disease-carrying allele. *P*_normal − supportive combination_ means the probability of the genotype combinations of the parents and embryos, based on which the site appears to come from the healthy allele. And *P*_neutral combination_ means the probability of the genotype combinations of the parents and embryos, based on which we cannot decide the allele origin for the embryo.

$$ {P}_{\mathrm{combination}}=\prod \limits_j{P}_{\mathrm{gt}\ \mathrm{of}\ \mathrm{sample}\ j\ \mathrm{in}\ \mathrm{the}\ \mathrm{combination}} $$

$$ {P}_{\mathrm{gt}\ \mathrm{of}\ \mathrm{sample}\ j}={P}_{\mathrm{gt}\mid \mathrm{all}\ \mathrm{read}\ \mathrm{data}}=\frac{P_{\mathrm{gt}}\times {P}_{\mathrm{all}\ \mathrm{read}\ \mathrm{data}\mid \mathrm{gt}}}{\sum \limits_{\mathrm{gt}}\left({P}_{\mathrm{gt}}\times {P}_{\mathrm{all}\ \mathrm{read}\ \mathrm{data}\mid \mathrm{gt}}\right)} $$

$$ {P}_{\mathrm{all}\ \mathrm{read}\ \mathrm{data}\mid \mathrm{gt}}={\Sigma}_{\mathrm{gt}\ \mathrm{after}\ \mathrm{amplification}}\left({P}_{\mathrm{gt}\ \mathrm{after}\ \mathrm{amplification}\mid \mathrm{gt}}\times {P}_{\mathrm{data}\mid \mathrm{gt}\ \mathrm{after}\ \mathrm{amplification}}\right) $$

$$ {P}_{\mathrm{data}\mid \mathrm{gt}\ \mathrm{after}\ \mathrm{amplification}}=\prod \limits_{\mathrm{read}}\left(\frac{P_{\mathrm{read}\mid \mathrm{haplotype}1}}{2}+\frac{P_{\mathrm{read}\mid \mathrm{haplotype}2}}{2}\right)\ \left[22\right] $$

Embryos with *P*_disease_ smaller than 10^−4^ are assumed to be “normal,” while those with *P*_disease_ between 10^−4^ and 0.1 are assumed to be “normal_risk.” Embryos with *P*_disease_ greater than 0.9 are assumed to be “disease-carrying” and those with *P*_disease_ between 0.9 and 0.6 are “disease_risk.” The embryos whose *P*_disease_ is between 0.1 and 0.6 are categorized as “risk.”

Error probability is the probability of making a wrong estimation of an embryo, which is 1− *P*_disease_ when we assume an embryo as a disease-carrying one and *P*_disease_ when we assume an embryo as a normal one.

The disease-carrying probability calculated via Bayesian approach was compared with the result of previous papers, which had already been validated by different platforms, including Sanger sequencing, aCGH and STR analyses [7, 17]. The transferred embryo was also validated to be disease-free in prenatal diagnosis by Sanger sequencing, karyotype, or SNP array by amniocentesis [7, 17].

### Phasing without proband sample

When a proband sample is absent, the disease-carrying allele is identified by grouping and phasing the genotypes of all embryos. First, the allele inherited from the disease-carrying parent is deduced for each embryo. Since these alleles are from the disease-carrying parent, it should be either disease-carrying allele or normal allele. The next step is to group these alleles into two classes according to the two kinds of genotypes at several sites. To group as many alleles as possible, we chose sites where the genotypes of most embryos, or most embryos and sperm samples, are specified. Finally, nucleotide composition is unified according to alleles in each class. The two unified alleles are the two alleles of the disease-carrying parent. The allele with causal mutation is the disease-carrying allele, while the allele without the causal mutation is the normal allele (Fig. 1c). To avoid genotype errors in embryos or disease-carrying parent, we discard those sites with more than one discordant sample, or those having the same genotype in two alleles.

All steps are detailed in a program online (https://github.com/XiongLuoxing/MARSALA). Once the raw sequencing files of volunteer family members are given, copy number variation (CNV) plot and linkage analyses results could be incorporated in the database automatically.

## Results

### Linkage analyses with proband sample

**Fig. 2:**
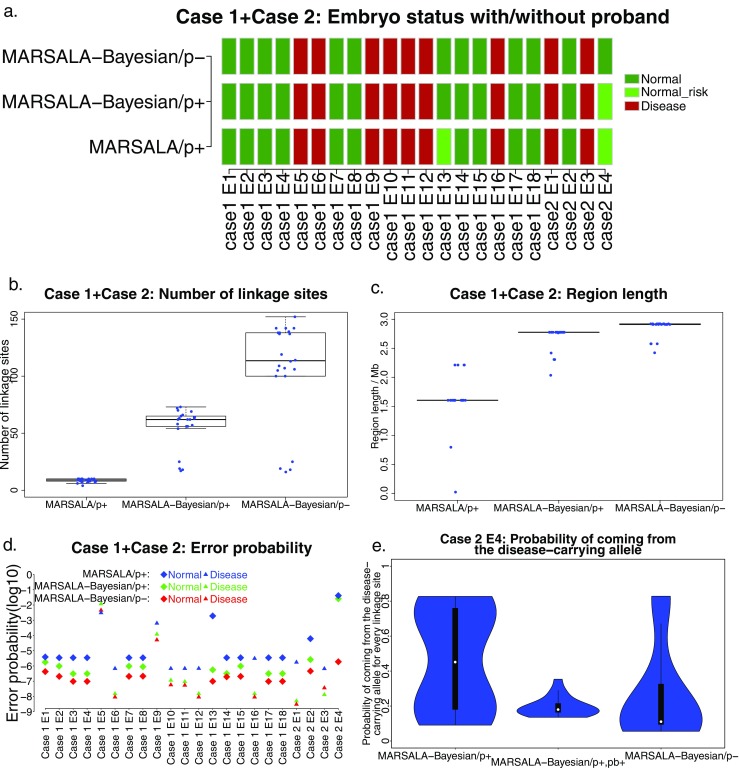
Linkage analyses output with Bayesian program for cases 1 and 2. **a** The disease-carrying status of every embryo in three modes. MARSALA/p+: Using the same ten sites as previous MARSALA analyses, the error probability was calculated for every embryo and embryo state was re-evaluated. MARSALA-Bayesian/p+: With proband sample, use all available sites to estimate embryo status with the Bayesian model. MARSALA-Bayesian/p−: Excludes the proband sample for analyses, evaluate embryo status with the Bayesian model. **b** Boxplot of linkage sites number used in the three modes. Outliers of MARSALA-Bayesian/p− and MARSALA-Bayesian/p+ modes are from case 2. **c** Boxplot of the length of linkage region for every embryo in the three modes. **d** Error probability calculated in the three modes. **e** Vioplot of the probabilities of coming from the disease-carrying allele for linkage sites of E4 in case 2 in three modes. MARSALA-Bayesian/p+,pb+: With both proband sample and polar bodies, evaluate embryo status using Bayesian model. The curve is rotated kernel density of the probabilities of coming from the disease-carrying allele for every linkage site. The central bar is boxplot

Compared with previous MARSALA analyses [7], the incorporation of the Bayesian program can in general achieve smaller error probability (Fig. 2a, d). To evaluate previous MARSALA analyses, the error probability was calculated for every embryo using Bayesian model with the same ten sites as the previous MARSALA analyses and the disease causal mutation site together. The embryo status was then re-estimated in this calculation mode, which is called MARSALA/proband+, i.e., MARSALA/p+. (Fig. 2a, Table S2).

In case 1, using MARSALA/p+, error probability of E13 is even larger than 10^−4^, so that it is estimated to be normal_risk under current criteria. We think that ten sites are not enough to deduce the disease-carrying status and avoid site selection bias. All available sites are used to estimate embryo status with the Bayesian model, which is called MARSALA-Bayesian/proband+, i.e., MARSALA-Bayesian/p+. With the incorporation of Bayesian model, the number of linkage sites is substantially increased from 10 to more than 60 (Fig. 2b) in a similar region (Fig. 2c). More linkage sites increased the accuracy of the linkage analyses and E13 can be classified as a normal embryo with 69 linkage sites in MARSALA-Bayesian/p+. Compared with MARSALA/p+, the error probability decreased for almost every embryo in case 1 with MARSALA-Bayesian/p+ (Fig. 2d). In case 1, embryo statuses are all correct and error probability of normal embryos ranges from 10^−6^ to 10^−7^ using Bayesian linkage analyses (Fig. 2a, d, Table S3).

In case 2, error probability by MARSALA-Bayesian/p+ was also reduced compared with that obtained with MARSALA/p+ (Fig. 2d, Table S2, Table S3). The number of linkage sites was increased with Bayesian model from 10 to 20 (Fig. 2b) in a similar region (Fig. 2c). Different from case 1, 60% of the flanking 3 Mb region in case 2 is masked as repeat region by repeat mask [24], which introduced an additional error due to mapping and SNP calling process. For E4, the error probability was larger than 10^−4^, both in MARSALA/p+ and MARSALA-Bayesian/p+; thus, it was estimated to be normal_risk (Fig. 2a). The linkage sites were limited in both MARSALA-Bayesian/p+ and MARSALA/p+, and in this embryo, near half of the sites appeared to come from the disease-carrying allele. Yet the embryo was normal according to PCR result of the disease causal mutation site (Fig. S2c); therefore, this embryo was finally estimated as “normal_risk.” This embryo had proven to be normal by other methods in previous MARSALA analyses, including Sanger sequencing of the PCR product and linkage analyses by polar bodies [7] (Fig. 2e, Fig. S2b). If polar bodies were also used to do linkage analysis, which is called MARSALA-Bayesian/p+,pb+, all sites were in strong support of coming from the normal allele (Fig. 2e), E4 can then be confidently estimated as normal (Fig. S2a, Table S4). Compared with the genotypes of embryos and polar bodies, those sites that seemed to come from the disease-carrying allele turned out to suffer from genotyping errors and were removed with MARSALA-Bayesian/p+,pb+.

In conclusion, the Bayesian model allows for more linkage sites and is free from site selection bias. In addition, site information is fully considered, making the error probability lower and making embryo status identification more accurate. More samples, like polar body, should be included to improve the accuracy of the analyses when the sample collection is possible, especially if the causal mutation is located in a repeat masked region of the genome.

### Linkage analyses without proband sample

Linkage analyses become a necessity in IVF when helping couples without proband sample. In this study, we have demonstrated that linkage analyses can be achieved without proband sample (MARSALA-Bayesian/p−) when no less than four embryos were sequenced and the causal mutation site has been amplified.

Incorporating Bayesian approach allows us to perform linkage analyses without proband sample in case 1 and case 2. As shown in Fig. 2a, disease-carrying statuses of all embryos were confirmed correct, including E4 in case 2 (Fig. 2a, Table S5). For all embryos, the number of linkage sites was further increased to about 120 (Fig. 2b) in similar linkage region (Fig. 2c) and error probability was actually smaller than that of linkage analyses with proband sample (Fig. 2d). The smallest error probability of normal embryos was decreased from 10^−6^ to 10^−8^ in both case 1 and case 2 (Fig. 2d). For E4 in case 2, we estimated it to be normal with low error probability in MARSALA-Bayesian/p−. This embryo was estimated as normal_risk in MARSALA/p+ and MARSALA-Bayesian/p+ due to several sites that appeared to come from the disease-carrying allele, which were caused by mapping errors. By comparing genotypes with other embryos, most of these sites that appeared to come from the disease-carrying allele turned out to have the wrong genotypes and thus filtered. And, more sites were found to come from the normal allele in MARSALA-Bayesian/p−. So we could make a correct evaluation of disease-carrying status of E4 in case 2 (MARSALA-Bayesian/p−, Fig. 2e).

In addition to case 1 and case 2, we performed linkage analyses without proband sample in case 3. Disease-carrying status of the disease from the mother was estimated and confirmed to be correct for each embryo (Table S6).

Our results demonstrate that linkage analyses performed with Bayesian offered better results than the commonly used with proband sample. In the process of grouping and phasing, genotypes of embryos were cross-validated, and genotype errors of most sites were efficiently identified and omitted. By omitting those errors in all embryos, the disease-carrying status can be correctly estimated with a much lower error probability (Fig. 2d).

### Linkage analyses with sperm and not proband sample

Linkage analyses without proband sample require a minimum of four embryos. In extreme cases when there are not enough embryos, sperm cells are an alternative if the father is the disease-carrying parent [17]. In case 3, we tested linkage analyses with sperm cells for each embryo. In this case, 6 embryos and 7 sperm cells were sequenced along with the parents’ genomic DNA.

**Fig. 3:**
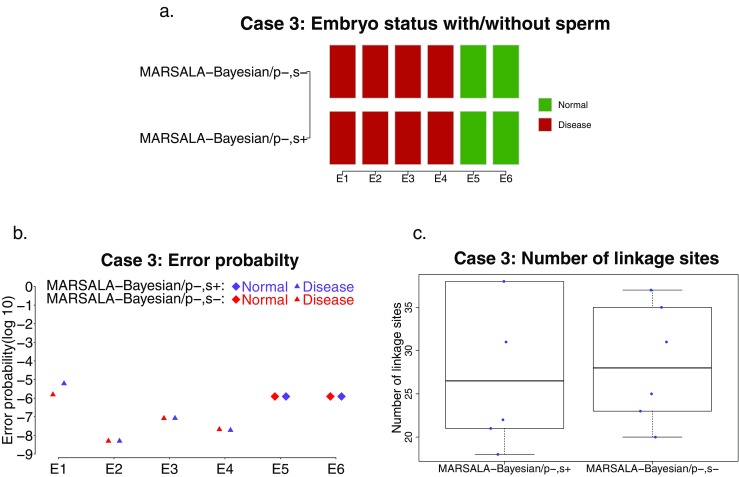
Compare linkage analyses with or without sperm. **a** The disease-carrying status of case 3 in two modes. MARSALA-Bayesian/p−,s+: When proband is unavailable, phase with 7 sperm cells and one embryo to estimate the disease-carrying status for the embryo. MARSALA-Bayesian/p−,s−: Without proband or sperm cells, phase with 6 embryos to estimate the embryo status. **b** Error probability of embryo status evaluation. **c** Boxplot of the number of linkage sites

We compared this mode (MARSALA-Bayesian/p−,s+) with the previously successful linkage analyses without proband sample or sperm (MARSALA-Bayesian/p−,s−). In MARSALA-Bayesian/p−,s+, only sperm cells were used to construct the disease-carrying allele and the normal allele, disease-carrying status was then deduced for each embryo. As for in MARSALA-Bayesian/p−,s−, sperm cells were excluded for analyses and all of the six embryos were used to construct haplotype and perform linkage analyses. Using sperm instead of embryo also allowed for correct deduction of all embryos’ statuses (Fig. 3a, Table S7, Table S8). The number of linkage sites and the error probability were comparable in these two modes, sperm and embryo (Fig. 3b–d).

Therefore, we suggest to sequence sperm cells when the father is the mutation carrier and there is less than 4 embryos. We have demonstrated here that linkage analyses with sperm cells could be as reliable as linkage analyses when more than three embryos are available.

## Discussion

In this study, Bayesian statistics model was used to complement with PCR results and linkage analyses from IVF cases previously published in MARSALA papers, and proven to increase the accuracy of embryo classification. Since false positives and false negatives in single-cell whole genome amplification is relatively high, the error probability of linkage analyses with few sites is still too high for IVF embryo selection. When single-cell WGA’s errors occur in the disease site, linkage analyses become the only method to determine the disease-carrying allele, leaving no alternative other than choosing the analyses sites manually. The Bayesian statistics method would then be of advantage since it is an automatic way to perform the SNV detection with high accuracy.

Our research also shows increased accuracy for linkage analyses in the absence of the commonly used proband sample. We have demonstrated that cross-validation between more samples improves accuracy, as cross-validation with more embryos, polar bodies, or sperm samples can efficiently remove genotyping errors. Although linkage analyses without proband sample has been reported [19] using an affected embryo as standard of affected allele, our method introduces cross-validation among all embryos to identify the affected allele. Using only an affected embryo may not be enough to construct the disease-carrying allele, particularly when the causal mutation is located in a repetitive region, as it is in case 2. In mode MARSALA-Bayesian/p+, one single proband sample is used to construct the disease-carrying allele and the genotyping errors make it difficult to assign a definite embryo status for E4 in case 2. However, in mode MARSALA-Bayesian/p−, several embryos are used to construct the disease-carrying allele and the embryo can be classified as normal. Therefore, using several samples to do phasing is necessary to avoid genotyping errors.

We propose that linkage analyses error in PGT-M could be significantly reduced from the conventional average of 0.3–0.4% [25] to 10^−6^–10^−4^% using the Bayesian program. The error after implementing Bayesian would depend on the lowest error probability of all embryos. The improved accuracy on embryo status determination by the Bayesian model can be explained by the incorporation of potential recombination events and/or genotyping errors in the program. With Bayesian application, the embryo with the lowest error probability is the best candidate for transfer. Indeed, with Bayesian, genotyping errors may become not so critical for linkage analyses, and linkage sites do not need rigidly more than 10 reads’ coverage, as is commonly practiced. Our research has shown that a coverage depth limit of 2 or 3 could multiply the number of linkage sites, which in return will provide more information on whether the allele is disease-carrying or normal. The more sites used, the lower error probability is achieved (Fig. S2b). With the maximum 30 sites used in Karyomapping [6], the error probability is 10^−4^%. Although the idea of integrating potential recombination events and genotyping errors had been reported [8, 9], we demonstrated here that choosing embryos by comparing error probability adds another key level to improve PGT-M accuracy.

The integration of recombination events in the Bayesian model is based on the assumption that recombination in a non-overlapping region is independent. Although some cases of recombination dependency have been reported, such as cross-over interference [26], we have not found better evidence or database describing a detailed and accurate recombination rate. But, if needed, we could easily integrate that into the proposed model.

Although we limited the Bayesian model to MALBAC amplified samples, we would like to point out that Bayesian could also be used with data from other genome amplification methods. We have not yet tested other methods due to the unsatisfactory quality of the data available, which is insufficient for our comparative studies. When the Bayesian model is applied to any data source, the parameters, especially allele dropout, false positives, and depth limit need to be adjusted before its wide clinical application.

In conclusion, the error probability of selecting healthy embryos for PGT-M based on linkage analyses has been quantified by using Bayesian statistics. In doing so, we are able to free the proband requirement in the linkage analysis. Although it is limited by cases where the causal mutation site cannot be amplified, or where the number of embryos is smaller than four and the disease-carrying parent is the mother, the Bayesian model presents tremendous advantages in improving the precision and simplification of the embryo selection in IVF.

## Electronic supplementary material

ESM 1 (DOCX 3595 kb)
