# Supplementary material for: Bayesian model for accurate MARSALA (mutated allele revealed by sequencing with aneuploidy and linkage analyses)
Source: J Assist Reprod Genet. 2019 Jun 11;36(6):1263–71. doi: 10.1007/s10815-019-01451-8 (PMC6602990; doi:10.1007/s10815-019-01451-8)
Supplement: Supplementary file 1 — (DOCX 3595 kb) [file 10815_2019_1451_MOESM1_ESM.docx]

Supplementary Online Material for

Bayesian approach for mutated allele revealed by sequencing with aneuploidy and linkage analysis(MARSALA)

# Methods

## SNV calling

The common procedures of calling SNP and phasing with proband is:

1. Using bwa-mem[28] to map fastq file against human reference and GATK^24^ to call SNP in pre-specified region, 1.5Mb upstream and downstream of disease site, and then removing those with low depth. The depth threshold could be user specified according to the amplification method, sequencing depth and location of causal mutation site. In the current cases, depth limit of proband and parents is 10. Depth limit of embryos is 0 in case1, 2 in case2 and multiplex PCR families. When disease site locates in repeat region or sequencing quality is not high enough, higher depth limit of the embryo is recommended.
2. Phasing between parents and embryos, inferring the allele passed from diseased parent to every embryo, i.e. inferring which nucleotide is passed from diseased parent to embryo at every linkage site(fig. S1). Genotype of the diseased parent has to be heterozygous to distinguish normal allele from abnormal one. As for embryo, there are 2 kinds of linkage sites. At some linkage sites, the genotype of the embryo is homozygous, the shared nucleotide between diseased parent and embryo is the passed nucleotide. At the rest, genotype of embryo is same as diseased parent yet different from the healthy parent. The passed nucleotide from diseased parent could then be specified after the nucleotide passed from the other parent if known.
3. If proband is given, phasing between proband and diseased parent and thus specifying the nucleotide composition of disease allele and normal allele. In this occasion, sites have to be heterozygous in diseased parent and homozygous in proband.

## CNV calling

CNV analysis process of the bayesian program is specific to MALBAC amplified data.

1. After mapping with bwa, CNV is called by freec[31]. In every embryo, chromosomes are marked if it has large CNV(30Mb) or most of the chromosome carries CNV.
2. Systems amplification bias of MALBAC is calculated from chromosomes unmarked with CNV of all embryos. And the bias is removed from all embryos amplified by MALBAC. CNV plot of all embryos in case1, case2 and case3 are presented in supplementary figures.

Of note, the range of system bias seems to be related to the microenvironment while amplification and varies from batches of experiment. Therefore, we recommend to set the experiment batch of embryos in input. Here, E5 and E13 are from different batches in case1. E4 is from a different batch in case2.

## Phasing with proband sample

For every embryo, phasing is to find the whether the allele inherited from the disease-carrying parent is the affected allele or not. It is composed of two steps. First, deduce what is the affected or normal allele in the disease-carrying parent, i.e. find the nucleotide composition at several sites near affected area of both alleles (fig. S1). As we need to distinguish affected allele from normal allele, those sites have to be heterozygous in disease-carrying parent. When probed sample is present, the nucleotide shared by proband sample and the disease-carrying parent is the nucleotide present in affected allele. So, proband sample has to be homozygous in those sites. Next, deduce what is the nucleotide inherited from disease-carrying parent to embryo at those sites near affected area (Fig S1). If the nucleotide is the same as the one shared by proband sample and disease-carrying parent, the site might come from the affected allele, and vice versa. Again, the shared the nucleotide between embryo and disease-carrying parent is the nucleotide inherited between them. Only for sites where the disease-carrying parent is heterozygous and either the embryo or the normal parent is homozygous can we specify the shared nucleotide.

# Reference.

[31]. Boeva, V., Popova, T., Bleakley, K., Chiche, P., Cappo, J., Schleiermacher, G., Janoueix-Lerosey, I., Delattre, O., & Barillot, E. Control-FREEC: A tool for assessing copy number and allelic content using next-generation sequencing data. Bioinformatics. 2012;28(3):423-425.

# Figures.

FigureS1

FigureS2

FigureS3

FigureS4

FigureS5

# Figure Legends.

Figure S1. Example of phasing. Adopted from Yan L. et al^11^.

Figure S2. Evaluation of embryo status in case2. a. Embryo status in case2. The mode MARSALA-Bayesian/p+,pb+ is provided in addition to fig. 2a. MARSALA-Bayesian/p+,pb+: With both proband sample and polar bodies, evaluate embryo status using Bayesian model. b. The probability of coming from the disease-carrying allele of all linkage sites in different modes for E4 in case2. c. The ratio of reads with/without causal mutation at the disease site in samples including proband sample(the born disease-carrying child), parents, polar bodies of E4 and E4 in case2.

Figure S3. CNV plot of case1 by the program.

Figure S4. CNV plot of case2 by the program.

Figure S5. CNV plot of case3 by the program.

# Tables.

Table S1．PCR primers close to the affected area.

| Case id | Forward Primer | Reverse primer |
| --- | --- | --- |
| case1 | GGGAGTATAATGAACTGCTCATGG | GGCTGTATGAGTGTGAGATACCTA |
| case2 | CACTGACTTTGCCAGCTATGA | GTGTGCTTGCTCATGTTGATG |
| case3 | CAGTGCCAGAAGAGCCAAG | AAGGTGCCCTTGAGGTTGTC |

Table S2. Report of linkage analysis by the program for case1 and case2 in MARSALA/p+.

| Case | ID | chr | left | right | disease supportive site | all site | disease supportive site ratio | P(disease) | status |
| --- | --- | --- | --- | --- | --- | --- | --- | --- | --- |
| case1 | E1 | chr11 | 43575766 | 45179706 | 1 | 11 | 0.0909 | 3.87E-06 | Normal |
| case1 | E2 | chr11 | 43575766 | 45179706 | 1 | 11 | 0.0909 | 3.46E-06 | Normal |
| case1 | E3 | chr11 | 43575766 | 45179706 | 1 | 11 | 0.0909 | 3.46E-06 | Normal |
| case1 | E4 | chr11 | 43575766 | 45179706 | 1 | 11 | 0.0909 | 3.46E-06 | Normal |
| case1 | E5 | chr11 | 43575766 | 45179706 | 10 | 10 | 1 | 9.97E-01 | Disease |
| case1 | E6 | chr11 | 43575766 | 45179706 | 10 | 11 | 0.9091 | 1.00E+00 | Disease |
| case1 | E7 | chr11 | 43575766 | 45179706 | 1 | 11 | 0.0909 | 3.46E-06 | Normal |
| case1 | E8 | chr11 | 43575766 | 45179706 | 1 | 11 | 0.0909 | 3.46E-06 | Normal |
| case1 | E9 | chr11 | 43575766 | 45179706 | 11 | 11 | 1 | 9.99E-01 | Disease |
| case1 | E10 | chr11 | 43575766 | 45179706 | 10 | 11 | 0.9091 | 1.00E+00 | Disease |
| case1 | E11 | chr11 | 43575766 | 45179706 | 10 | 11 | 0.9091 | 1.00E+00 | Disease |
| case1 | E12 | chr11 | 43575766 | 45179706 | 10 | 11 | 0.9091 | 1.00E+00 | Disease |
| case1 | E13 | chr11 | 43575766 | 45179706 | 5 | 11 | 0.4545 | 1.92E-03 | Normal_risk |
| case1 | E14 | chr11 | 43575766 | 45179706 | 1 | 11 | 0.0909 | 3.46E-06 | Normal |
| case1 | E15 | chr11 | 43575766 | 45179706 | 1 | 11 | 0.0909 | 3.46E-06 | Normal |
| case1 | E16 | chr11 | 43575766 | 45179706 | 9 | 10 | 0.9 | 1.00E+00 | Disease |
| case1 | E17 | chr11 | 43575766 | 45179706 | 1 | 11 | 0.0909 | 3.46E-06 | Normal |
| case1 | E18 | chr11 | 43575766 | 45179706 | 1 | 11 | 0.0909 | 3.46E-06 | Normal |
| case2 | E1 | chrX | 68162230 | 70374204 | 9 | 10 | 0.9 | 1.00E+00 | Disease |
| case2 | E2 | chrX | 68162230 | 70374204 | 0 | 9 | 0 | 6.14E-05 | Normal |
| case2 | E3 | chrX | 68162230 | 70374204 | 10 | 10 | 1 | 1.00E+00 | Disease |
| case2 | E4 | chrX | 68535158 | 70374204 | 3 | 6 | 0.5 | 4.13E-02 | Normal_risk |

Table S3. Report of linkage analysis by the program for case1 and case2 in MARSALA-Bayesian/p+.

| Case | ID | chr | left | right | disease supportive site | all site | disease supportive site ratio | P(disease) | status |
| --- | --- | --- | --- | --- | --- | --- | --- | --- | --- |
| case1 | E1 | chr11 | 42725806 | 45502046 | 6 | 56 | 0.1071 | 1.77E-06 | Normal |
| case1 | E2 | chr11 | 42725806 | 45502046 | 6 | 58 | 0.1034 | 9.68E-07 | Normal |
| case1 | E3 | chr11 | 42725806 | 45502046 | 7 | 62 | 0.1129 | 3.08E-07 | Normal |
| case1 | E4 | chr11 | 42725806 | 45502046 | 6 | 64 | 0.0938 | 3.11E-07 | Normal |
| case1 | E5 | chr11 | 42725806 | 45502046 | 58 | 65 | 0.8923 | 9.88E-01 | Disease |
| case1 | E6 | chr11 | 42725806 | 45502046 | 70 | 73 | 0.9589 | 1.00E+00 | Disease |
| case1 | E7 | chr11 | 42725806 | 45502046 | 4 | 56 | 0.0714 | 9.60E-07 | Normal |
| case1 | E8 | chr11 | 42725806 | 45502046 | 4 | 54 | 0.0741 | 8.92E-07 | Normal |
| case1 | E9 | chr11 | 42725806 | 45502046 | 60 | 63 | 0.9524 | 1.00E+00 | Disease |
| case1 | E10 | chr11 | 42725806 | 45502046 | 60 | 66 | 0.9091 | 1.00E+00 | Disease |
| case1 | E11 | chr11 | 42725806 | 45502046 | 57 | 62 | 0.9194 | 1.00E+00 | Disease |
| case1 | E12 | chr11 | 42725806 | 45502046 | 70 | 72 | 0.9722 | 1.00E+00 | Disease |
| case1 | E13 | chr11 | 42725806 | 45502046 | 24 | 69 | 0.3478 | 5.53E-07 | Normal |
| case1 | E14 | chr11 | 42725806 | 45502046 | 6 | 64 | 0.0938 | 3.13E-07 | Normal |
| case1 | E15 | chr11 | 42725806 | 45502046 | 7 | 57 | 0.1228 | 9.69E-07 | Normal |
| case1 | E16 | chr11 | 42725806 | 45502046 | 68 | 70 | 0.9714 | 1.00E+00 | Disease |
| case1 | E17 | chr11 | 42725806 | 45502046 | 5 | 63 | 0.0794 | 3.14E-07 | Normal |
| case1 | E18 | chr11 | 42725806 | 45491655 | 5 | 62 | 0.0806 | 3.14E-07 | Normal |
| case2 | E1 | chrX | 68049982 | 70468754 | 24 | 25 | 0.96 | 1.00E+00 | Disease |
| case2 | E2 | chrX | 68162230 | 70468754 | 2 | 19 | 0.1053 | 2.61E-06 | Normal |
| case2 | E3 | chrX | 68162230 | 70468754 | 17 | 17 | 1 | 1.00E+00 | Disease |
| case2 | E4 | chrX | 68433662 | 70468754 | 9 | 18 | 0.5 | 2.57E-02 | Normal_risk |

Note. P(disease) in linkage analysis report file equals to error probability when the embryo is diagnosed as normal and equals to 1-error probability when it is diagnosed as disease carrying.

Table S4. Report of linkage analysis by the program for case2 in MARSALA-Bayesian/p+,pb+.

| Case | ID | chr | left | right | disease supportive sites | all site | disease supportive site ratio | P(disease) | status |
| --- | --- | --- | --- | --- | --- | --- | --- | --- | --- |
| case2 | PB1 | chrX | 68162230 | 70445407 | 19 | 20 | 0.95 | 1.00E+00 | Disease |
| case2 | PB2 | chrX | 68049982 | 70468754 | 0 | 25 | 0 | 6.50E-07 | Normal |
| case2 | PB3 | chrX | 68162230 | 70468754 | 18 | 18 | 1 | 1.00E+00 | Disease |
| case2 | PB4 | chrX | 68162230 | 70374204 | 0 | 19 | 0 | 6.17E-06 | Normal |

Table S5. Report of linkage analysis by the program for case1 and case2 in MARSALA-Bayesian/p-.

| Case | ID | chr | left | right | disease supportive site | all site | disease supportive site ratio | P(disease) | status |
| --- | --- | --- | --- | --- | --- | --- | --- | --- | --- |
| case1 | E1 | chr11 | 42699672 | 45611238 | 4 | 106 | 0.0377 | 4.24E-07 | Normal |
| case1 | E2 | chr11 | 42699672 | 45621378 | 2 | 107 | 0.0187 | 2.06E-07 | Normal |
| case1 | E3 | chr11 | 42699672 | 45621378 | 6 | 139 | 0.0432 | 9.64E-08 | Normal |
| case1 | E4 | chr11 | 42699672 | 45621378 | 2 | 142 | 0.0141 | 9.63E-08 | Normal |
| case1 | E5 | chr11 | 42699672 | 45611238 | 93 | 119 | 0.7815 | 9.95E-01 | Disease |
| case1 | E6 | chr11 | 42699672 | 45611238 | 136 | 138 | 0.9855 | 1.00E+00 | Disease |
| case1 | E7 | chr11 | 42699672 | 45611238 | 2 | 109 | 0.0183 | 2.06E-07 | Normal |
| case1 | E8 | chr11 | 42699672 | 45621378 | 3 | 113 | 0.0265 | 2.12E-07 | Normal |
| case1 | E9 | chr11 | 42699672 | 45611238 | 93 | 100 | 0.93 | 1.00E+00 | Disease |
| case1 | E10 | chr11 | 42699672 | 45611238 | 95 | 100 | 0.95 | 1.00E+00 | Disease |
| case1 | E11 | chr11 | 42699672 | 45621378 | 97 | 105 | 0.9238 | 1.00E+00 | Disease |
| case1 | E12 | chr11 | 42699672 | 45611238 | 133 | 137 | 0.9708 | 1.00E+00 | Disease |
| case1 | E13 | chr11 | 42699672 | 45621378 | 35 | 152 | 0.2303 | 9.81E-08 | Normal |
| case1 | E14 | chr11 | 42699672 | 45621378 | 5 | 142 | 0.0352 | 1.92E-07 | Normal |
| case1 | E15 | chr11 | 42699672 | 45621378 | 5 | 114 | 0.0439 | 2.06E-07 | Normal |
| case1 | E16 | chr11 | 42699672 | 45611238 | 136 | 138 | 0.9855 | 1.00E+00 | Disease |
| case1 | E17 | chr11 | 42699672 | 45621378 | 3 | 142 | 0.0211 | 9.63E-08 | Normal |
| case1 | E18 | chr11 | 42699672 | 45611238 | 1 | 137 | 0.0073 | 9.72E-08 | Normal |
| case2 | E1 | chrX | 67890871 | 70468754 | 22 | 25 | 0.88 | 1.00E+00 | Disease |
| case2 | E2 | chrX | 67890871 | 70468754 | 0 | 19 | 0 | 4.56E-07 | Normal |
| case2 | E3 | chrX | 68045863 | 70468754 | 16 | 16 | 1 | 1.00E+00 | Disease |
| case2 | E4 | chrX | 67890871 | 70468754 | 3 | 18 | 0.1667 | 1.88E-06 | Normal |

Table S6. Report of linkage analysis of mother side by the program for case3 in MARSALA-Bayesian/p-. Embryo statuss are all correctly estimated of the disease from the mother.

| case | ID | chr | left | right | disease supportive site | all site | disease supportive site ratio | P(disease) | status |
| --- | --- | --- | --- | --- | --- | --- | --- | --- | --- |
| case3 | E1 | chr11 | 3932805 | 6667190 | 0 | 53 | 0 | 3.78E-08 | Normal |
| case3 | E2 | chr11 | 3967171 | 6720611 | 40 | 53 | 0.7547 | 1.00E+00 | Disease |
| case3 | E3 | chr11 | 3932805 | 6720611 | 0 | 65 | 0 | 4.89E-09 | Normal |
| case3 | E4 | chr11 | 3932805 | 6720611 | 0 | 67 | 0 | 9.75E-09 | Normal |
| case3 | E5 | chr11 | 3932805 | 6667190 | 4 | 65 | 0.0615 | 7.82E-08 | Normal |
| case3 | E6 | chr11 | 3932805 | 6667190 | 3 | 67 | 0.0448 | 8.01E-08 | Normal |

Table S7. Report of linkage analysis by the program for case3 in MARSALA-Bayesian/p-,s+

| case | ID | chr | left | right | disease supportive site | all site | disease supportive site ratio | P(disease) | status |
| --- | --- | --- | --- | --- | --- | --- | --- | --- | --- |
| case3 | E1 | chr11 | 4391231 | 6578004 | 18 | 22 | 0.8182 | 1.00E+00 | Disease |
| case3 | E2 | chr11 | 4179324 | 6729693 | 29 | 31 | 0.9355 | 1.00E+00 | Disease |
| case3 | E3 | chr11 | 4623106 | 6729693 | 17 | 18 | 0.9444 | 1.00E+00 | Disease |
| case3 | E4 | chr11 | 4179324 | 6729693 | 20 | 21 | 0.9524 | 1.00E+00 | Disease |
| case3 | E5 | chr11 | 4391231 | 6585007 | 1 | 38 | 0.0263 | 1.24E-06 | Normal |
| case3 | E6 | chr11 | 4391231 | 6585007 | 1 | 38 | 0.0263 | 1.24E-06 | Normal |

Table S8. Report of linkage analysis by the program for case3 in MARSALA-Bayesian/p-,s-

| case | ID | chr | left | right | disease supportive site | all site | disease supportive site ratio | P(disease) | status |
| --- | --- | --- | --- | --- | --- | --- | --- | --- | --- |
| case3 | E1 | chr11 | 4391231 | 6578004 | 22 | 25 | 0.88 | 1.00E+00 | Disease |
| case3 | E2 | chr11 | 4179324 | 6729693 | 31 | 31 | 1 | 1.00E+00 | Disease |
| case3 | E3 | chr11 | 4623106 | 6729693 | 19 | 20 | 0.95 | 1.00E+00 | Disease |
| case3 | E4 | chr11 | 4179324 | 6729693 | 22 | 23 | 0.9565 | 1.00E+00 | Disease |
| case3 | E5 | chr11 | 4391231 | 6585007 | 3 | 35 | 0.0857 | 1.24E-06 | Normal |
| case3 | E6 | chr11 | 4391231 | 6585007 | 3 | 37 | 0.0811 | 1.24E-06 | Normal |
